# Role of breastfeeding in disease prevention

**DOI:** 10.1111/1751-7915.14520

**Published:** 2024-06-30

**Authors:** Andrea C. Masi, Christopher J. Stewart

**Affiliations:** ^1^ Translational and Clinical Research Institute Newcastle University Newcastle upon Tyne UK

## Abstract

Human milk provides the infant with many bioactive factors, including immunomodulating components, antimicrobials and prebiotics, which modulate the infant microbiome and immune system maturation. As a result, breastfeeding can impact infant health from infancy, through adolescence, and into adulthood. From protecting the infant from infections, to reducing the risk of obesity, type 1 diabetes and childhood leukaemia, many positive health outcomes are observed in infants receiving breastmilk. For the mother, breastfeeding protects against postpartum bleeding and depression, increases weight loss, and long‐term lowers the risk of type 2 diabetes, breast and ovarian cancer, and cardiovascular diseases. Beyond infants and mothers, the wider society is also impacted because of avoidable costs relating to morbidity and mortality derived from a lack of human milk exposure. In this review, Medline was used to search for relevant articles to discuss the health benefits of breastfeeding and its societal impact before exploring future recommendations to enhance our understanding of the mechanisms behind breastfeeding's positive effects and promote breastfeeding on a global scale.

## Graphical abstract

Breastfeeding provides many benefits to both infants and mothers. Breastfed infants have a lower risk of infection and, later in life, are less likely to develop obesity, type 1 diabetes and childhood leukaemia. Breastfeeding protects the mother from postpartum bleeding and depression, type 2 diabetes, ovarian and breast cancer.

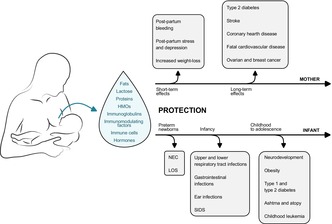

## INTRODUCTION

Human milk contains the optimal nutritional composition for infant physical and neurological growth, along with important bioactive components, including immunomodulatory factors, growth hormones and a combination of pro‐ and pre‐biotics (Perrella et al., 2021).

Lactose and lipids are the most abundant solid components making up human milk, with lactose being a crucial source of energy for infants and fats also providing energy and sustaining brain development, cell membrane structure, absorption of fat‐soluble vitamins and immune function (certain fats exhibit antimicrobial and anti‐inflammatory characteristics) (Ballard & Morrow, 2013; Reniker et al., 2023). Human milk oligosaccharides (HMOs) represent the third most abundant solid component. These are complex sugars indigestible to infants and are postulated to have varied activities, including shaping the infant gut microbiome (i.e. prebiotics) (Berger et al., 2020), acting as anti‐adhesive antimicrobials (thus protecting from infections) (Zuurveld et al., 2020), and directly modulating the immune system (Eiwegger et al., 2004) and intestinal epithelium barrier function (Natividad et al., 2020).

Proteins play a multitude of vital roles in growth, development and overall health, and more than 400 different proteins are present in human milk covering different functions (Donovan, 2019). Proteins aid infant nutrition and growth by providing essential amino acids necessary for the development of various tissues and organs (Donovan, 2019). Among human milk proteins are immunoglobulins, with secretory IgA being the most abundant and followed by IgG and IgM (Atyeo & Alter, 2021). These, together with other antimicrobial proteins such as lactoferrin and lysozyme, protect the infant against infections, especially in the first months of life when the immune system is immature and in the process of being educated by the microbes it encounters after birth (Reniker et al., 2023).

Mother's immune cells are also transferred to the infant through breastfeeding, including macrophages, T cells, lymphocytes, and stem cells, which help support the infant's developing immune system (Ballard & Morrow, 2013). Other factors such as hormones are also present in human milk and might have long‐term effects, including metabolic health through leptin, ghrelin, adiponectin, and insulin‐like growth factor 1 (Savino et al., 2009).

An important caveat of most studies discussed in this review is the lack of granular information relating to whether human milk feeding was direct at the breast or by expressed milk. While expression, storage, and subsequent thawing and warming of human milk have an impact on the activity of some of its components (Stinson et al., 2024), there is a paucity of information on how such practices could impact health outcomes, and further studies will be needed to fill this knowledge gap.

## HUMAN MILK AND INFANT GUT MICROBIOME COMPOSITION

Many human milk components shape the infant intestinal microbiome composition, including HMOs, fats, and immune‐modulatory molecules such as lysozyme, lactoferrin, and secreted IgA (sIgA) (Granger et al., 2021). HMOs are among the most studied; they act as prebiotics and can be digested by selected bacteria that are considered beneficial to the infant, mainly belonging to the *Bifidobacterium* genus but also by others such as *Bacteroides* spp. (Masi & Stewart, 2022). Human milk has also its own microbial community that can directly seed the infant gut microbiome, providing the infant with pioneer colonisers (Bogaert et al., 2023). As well as promoting colonisation directly and indirectly, human milk provides the infant with sIgA, which can bind to pathobionts to prevent their interaction with the intestinal epithelium (Donald et al., 2022). Furthermore, lysozyme and lactoferrin have important antimicrobial properties that further shape the infant intestinal microbiome (Granger et al., 2021).

Modulating the infant gut microbiome composition is one of the mechanisms through which human milk feeding may exert positive health outcomes. By favouring the growth of beneficial bacteria, human milk protects the infant from the colonisation of pathobionts preventing infections (Masi & Stewart, 2022). The microbial community in the first months of life is also involved in educating the infant's immune system and metabolism and has been associated with long‐term health outcomes, including a reduced risk of developing type 1 diabetes (Vatanen et al., 2018) and allergy (Abrahamsson et al., 2012). The bacterial community in the gut will also have a broad impact on the host beyond the gastrointestinal tract and immune systems through the production of functional metabolites that can translocate to the blood and exert systemic effects. For example, short‐chain fatty acids, that are produced by bacteria after the metabolism of certain dietary components such as HMOs, have been shown to regulate lipid metabolism and glucose homeostasis (Morrison & Preston, 2016).

Before further discussing the health benefits of breastfeeding, it has to be noted that a mother's health status has been demonstrated to alter the human milk composition. For instance, mother's BMI can influence the milk microbiome (Cortés‐Macías et al., 2021), macronutrient composition (Daniel et al., 2021) and overall amounts of the various bioactive components (Saben et al., 2020). This should not discourage the promotion of breastfeeding among women, and more studies are needed to understand how such alterations might affect the infant's health, and potentially define breastfeeding guidelines or targeted interventions tailored to each subgroup.

## DISEASE PROTECTION IN INFANCY

Human milk does not only affect gut health; its effects extend to neurodevelopment and overall health. Starting with the most vulnerable babies in the preterm population, human milk feeding reduces the risk of necrotising enterocolitis (Meinzen‐Derr et al., 2009) and late‐onset sepsis (Patel et al., 2013), which together account for the highest number of fatalities in preterm infants, as well as respiratory symptoms (Kim et al., 2019). In all infants, breastfeeding is associated with decreased all‐cause mortality and infection‐related mortality with a dose‐response correlation and up to 2 years of age (Sankar et al., 2015). Time of initiation of human milk feeding is important, with higher mortality observed in infants who initiated breastfeeding 24 hours after birth compared to infants who initiated human milk feeding earlier (Debes et al., 2013; Smith et al., 2017). Among the morbidities for which human milk gives protection, we can find various infections including upper and lower respiratory tract (e.g., pneumonia, bronchiolitis), gastrointestinal (e.g., diarrhoea), and ear infections (e.g., acute otitis media), as well as septicaemia (Debes et al., 2013; Hossain & Mihrshahi, 2022), and such beneficial effects are reported for both developed (Duijts et al., 2009; Ware et al., 2023) and underdeveloped countries (Abdulla et al., 2022). This likely relates to the presence of various bioactive components that include antibodies, immune cells, antimicrobial molecules, and a mix of probiotics and prebiotics, as described in the previous section.

While the mechanisms are still unclear, recent work has shown that breastfeeding protects new‐borns against Sudden Infant Death Syndrome (SIDS) (Vennemann et al., 2009). The most recent meta‐analysis on the subject found that such protection might occur only when the baby is breastfed for at least 2 months, with an increase in protection observed with longer duration in breastfeeding (Thompson et al., 2017). Infants who are breastfed have a more stable sleep pattern and show an improved ability to awaken from deep sleep, potentially explaining human milk protection from SIDS (Horne et al., 2004).

## LONG‐TERM HEALTH BENEFITS

The health benefits of receiving breastmilk in infancy extend into childhood and adulthood and include neurodevelopment, metabolic health and cancer prevention.

Studies over the years have focused on the relationship between breastfeeding and developmental outcomes and intelligence test performance (Horta et al., 2015a). Despite multiple studies reporting a positive effect of breastfeeding on neurodevelopment, not all of them have accounted for potential confounders (Bar et al., 2016; Horta et al., 2018). Indeed, most studies have been conducted in high‐income countries, in which longer breastfeeding is usually found in families with higher socioeconomic status, a factor that is also associated with better performance in intelligence tests (Horta et al., 2018). Moreover, mothers who are able to sustain breastfeeding are also more likely to engage in activities that stimulate their children at home, further improving the child's neurodevelopment (Horta et al., 2018). Various meta‐analyses have integrated the results from multiple studies by also filtering for data quality to control for confounding factors (Bar et al., 2016; Horta et al., 2015a, 2018). Not surprisingly, a smaller benefit of breastfeeding was reported when the outcome was adjusted for maternal IQ (Horta et al., 2015a). However, integration of studies with high‐quality data confirmed the positive correlation between breastfeeding and performance in intelligence tests (Horta et al., 2015a). Results from a trial in Belarus further support this positive association, where clinics randomised to promote breastfeeding showed longer durations of both total and exclusive breastfeeding, and the children belonging to this group exhibited better performance in intelligence tests at 6.5 years of age (Kramer et al., 2008). More recent studies on the association between breastfeeding and increased IQ in children further support the positive impact this practice has on neurodevelopment in the case of both term (Plunkett et al., 2021; Strøm et al., 2019) and preterm births (Belfort et al., 2022; Rodrigues et al., 2022), and in children at higher risk of developing autism spectrum disorders (Punatar et al., 2024).

Further supporting the effect breastfeeding has on neurodevelopment, multiple studies have reported different brain composition in infants who were breastfed compared to never breastfeeding or depending on longer breastfeeding duration, with impacts on grey matter (Belfort et al., 2016; Ou et al., 2016) and hippocampal volumes (Belfort et al., 2016), as well as brain activation (Ou et al., 2016) and cortical thickness (Grevet et al., 2023). One of the proposed mechanisms for the enhanced neurodevelopment of breastfed children is mediated by polyunsaturated fatty acids (PUFAs) found in human milk. The membrane of brain cells is particularly rich in long‐chain PUFAs, which cannot be produced by the infant who then relies on the diet to accumulate the necessary amount for brain development (Martinat et al., 2021). Moreover, PUFAs are also critical precursors for signalling molecules that target the brain among other organs (Martinat et al., 2021). Bernard et al. investigated PUFAs’ concentration in colostrum in relation to the child's IQ at 5–6 years of age from the EDEN cohort (Bernard et al., 2017). They found that colostrum concentrations of arachidonic acid and 3‐long‐chain PUFA were associated with children's IQ, with children receiving high levels of these two fatty acids in colostrum showing the highest IQ scores (Bernard et al., 2017). Notably, long‐chain PUFA supplementation of infant formula was not associated with neurodevelopment at 3.5 years of age in children from a French cohort (Martinot et al., 2022). HMOs have also been proposed to have a role in the infant's neurodevelopment not only by providing sialic acid, key to ganglioside formation and myelination, but also through the microbiome fermentation and release of SCFAs that can enter the nervous system and influence its gene expression (Berger et al., 2023). The current data suggests that various human milk components might act synergistically to enhance neurodevelopment and further studies are needed.

Long‐term breastfeeding has been linked to a lower risk of certain chronic conditions such as obesity, diabetes, asthma, and allergies later in life (Davis, 2001; Kelishadi & Farajian, 2014). Research suggests that breastfeeding is associated with a decreased risk of obesity from early childhood through to adolescence, potentially due to the self‐regulation of infant feeding patterns and the unique composition of human milk (Moreno et al., 2011). Studies finding such correlation included the one on a Scottish cohort of 32,200 children aged ~3.5 years (Armstrong & Reilly, 2002), a German cohort, including 9357 5–6 years old children (von Kries et al., 1999), and a US cohort of 15,341 adolescents aged 9–14 years old (Gillman et al., 2001). All studies mentioned adjusted for confounding factors such as socioeconomic status, and a dose‐response effect was observed, with prolonged breastfeeding correlated with lower rates of obesity (Gillman et al., 2001; Harder et al., 2005; von Kries et al., 1999). Among the potential factors decreasing the risk of obesity, we have the hormonal content of human milk, such as leptin, which may influence the regulation of appetite and energy balance (Palou et al., 2018). By establishing healthy dietary patterns early in life, breastfeeding can contribute to a reduced risk of obesity and related metabolic disorders later in childhood and adulthood (Moreno et al., 2011).

Breastfeeding has been associated also with protection against both type 1 (Group, 2002; Verge et al., 1994; Virtanen et al., 1992) and type 2 diabetes (Owen et al., 2006) (T1D and T2D respectively). For type 1 diabetes, the results reported are conflicting and are potentially influenced by the recall bias affecting retrospective studies, which make up most of the published research on the subject (Cardwell et al., 2012). Prospective studies from the past 10 years also report conflicting results. The MIDIA 2014 study, which followed up 908 Norwegian children with increased genetic susceptibility, found that any breastfeeding for 12 months or longer was associated with protection against T1D development (Lund‐Blix et al., 2014). In 2017 results from the Norwegian MoBa and Danish DNBC studies were published which together included over 150 k children. They found that infants who were never breastfed had a two‐fold increased risk of developing T1D, which was independent of the duration of breastfeeding (Lund‐Blix et al., 2017). In contrast, no protective association between breastfeeding and T1D development was found in the prospective Finnish study by Hakola et al., which included 5915 new‐borns at high risk of the disease (Hakola et al., 2017), and in the TEDDY study of 8676 high‐risk children from United States, Finland, Germany, and Sweden (Hummel et al., 2021). Protection against the development of T2D has also been reported. In Pima Indians and in the Native Canadian population, breastfeeding has been found to have a protective effect (Pettitt et al., 1997; Young et al., 2002). A correlation between lack of breastfeeding and impaired glucose tolerance has also been observed (Martin et al., 2005; Ravelli et al., 2000). Integrating multiple studies, two separate meta‐analyses found that early breastfeeding was associated with T2D protection (Horta et al., 2015b; Owen et al., 2006), and this is potentially linked to the lower obesity rate in individuals who were breastfed. Early‐life gut microbiome composition has been associated with an increased risk of obesity and T1D (Dedrick et al., 2020), potentially representing one of the multiple ways human milk shapes health beyond infancy. However, association does not mean causation and further studies are needed to disentangle the mechanisms through which breastfeeding might prevent such diseases.

As reported for the other correlations between breastfeeding and disease development, protection from asthma and atopy development is controversial (Nuzzi et al., 2021). While some studies report a protective effect (Dogaru et al., 2014; Hummel et al., 2021; Mimouni Bloch et al., 2002), others observed no impact of breastfeeding on allergy development, and some found an increased risk (Giwercman et al., 2010). Many studies relied on recall of breastfeeding information and did not correct for various confounders, including family history of atopy, which might influence the quality of the data (Nuzzi et al., 2021). Meta‐analyses have tried to evaluate the potential protective effect exerted by breastfeeding on allergic diseases; however, the results are inconclusive. Lodge et al. (2015) reported that breastfeeding might protect against asthma in children of 5–18 years of age and found a weaker protective effect against eczema in children ≤2 years of age and allergic rhinitis in ≤5 years of age (Lodge et al., 2015). No protection was found against food allergies. The authors commented that the quality of the studies included was considered low. Moreover, a bigger effect was observed in early life, potentially reflecting the protection from viral infections that can manifest with wheezing, and the greater protection in middle/low‐income counties is also supportive of this theory (Lodge et al., 2015).

An additional disease breastfeeding might help prevent is childhood leukaemia. Some differences were reported between studies specifically in relation to the months of breastfeeding needed to exert a protection. For instance, Shu et al. (1999) show that ever being breastfed was correlated with a 21% lower risk of developing childhood acute leukaemia compared to never being breastfed (Shu et al., 1999). However, more recent studies reported that a 20% lower risk of disease was observed when the infant was breastfed for at least 6 months, while ever versus never breastfed was associated with a 9% lower risk (Amitay & Keinan‐Boker, 2015). A recent meta‐analysis observed a non‐linear dose–response effect, with the lowest risk found at a breastfeeding duration of 9.6 months coinciding to 44% protection, while a 23% decrease in risk of developing leukaemia was reported for ever versus no/occasional breastfeeding and also a 23% lower risk for longest compared to shortest breastfeeding duration (Su et al., 2021).

## MATERNAL HEALTH BENEFITS

Beyond their infant, breastfeeding has also been associated with maternal health (Dieterich et al., 2013). Evidence shows that breastfeeding and skin‐to‐skin contact after birth help with postpartum recovery by inducing the release of oxytocin in the mother, which can contribute to reduced postpartum bleeding and help the uterus to return to its pre‐pregnancy size more quickly (Almutairi et al., 2021; Saxton et al., 2015). Breastfeeding has been associated with lower post‐partum depression and stress, with a dose–response effect being observed (Pope & Mazmanian, 2016). Mothers who practiced mixed breastfeeding showed a higher perceived stress and postpartum depression compared to mothers who exclusively breastfed (Gila‐Díaz et al., 2020; Thome et al., 2006). A possible reciprocal relationship has been proposed. A study by Hamdan and Tamim reported breastfeeding at 2 months postpartum was associated to lower depression at 4 months postpartum, and at the same time lower breastfeeding rate at 4 months was observed in mother suffering from postpartum depression at 2 months (Hamdan & Tamim, 2012). Further supporting this hypothesis, Figueiredo et al. found that depression scores in the third trimester were negatively associated with exclusive breastfeeding duration, and a decrease in depression scores was found from birth to 3 months postpartum in women who exclusively breastfed for longer than 3 months (Figueiredo et al., 2014). Notably, it is difficult to disentangle the correlation between breastfeeding and depression as maternal mental health challenges might lead to early cessation of breastfeeding (Pope & Mazmanian, 2016). Furthermore, correlation does not equal causation, and mothers may feel more depressed if, despite their best efforts, they have been unable to breastfeed their infant. This is amplified in the era of unregulated internet forums, social media posts and print media criticising mothers for not breastfeeding or presenting negative social attitudes towards breastfeeding (Merritt et al., 2023).

Pregnancy leads to various changes in the metabolic status of the mother and is also associated with long‐term weight gain (Dieterich et al., 2013). Multiple studies have reported enhanced weight loss in mothers who breastfed compared to mothers who formula‐fed their new‐borns, with a potential dose‐response relationship, where greater weight loss was correlated with exclusivity and duration of breastfeeding (Baker et al., 2008; Dewey et al., 1993; Jarlenski et al., 2014). Reduction in the risk of developing T2D and cardiovascular risk in the mother might also be a consequence of breastfeeding (Pinho‐Gomes et al., 2021; Tschiderer et al., 2022). A recent meta‐analysis has integrated the data from 16 studies finding a 27% lower risk of developing T2D in mothers who breastfed compared to mothers who never breastfed (Pinho‐Gomes et al., 2021). Women who developed gestational diabetes mellitus might experience a higher risk reduction, and a dose‐response effect was also observed, with each additional month of lactation being associated with an extra 1% lower risk of disease development (Pinho‐Gomes et al., 2021). Similarly, a meta‐analysis investigating the effect of breastfeeding on cardiovascular disease outcomes found protection exerted by this practice against stroke, coronary heart disease, and fatal cardiovascular disease (Tschiderer et al., 2022), for which dose‐response has been suggested (Schwarz et al., 2009). A potential explanation for such protection is described with the ‘Reset Hypothesis’ suggested by Stuebe and Rich‐Edwards (Stuebe & Rich‐Edwards, 2009). During pregnancy, a woman's metabolism changes to support the foetus growth and store energy for future lactation, resulting in the accumulation of visceral fat, insulin resistance and production, and circulating lipid levels (Stuebe & Rich‐Edwards, 2009). Animal studies have shown that lactation induces the uptake of the stored lipids in the mammary tissue where they are transferred into the milk (Hamosh et al., 1970), and the number (Moore & Brasel, 1984), dimension (Steingrimsdottir et al., 1980) and distribution (Moore et al., 1984) of fat cells were also influenced by lactation status with a positive health effect. Stuebe and Rich‐Edwards suggest that similar physiological changes happen in lactating women, potentially explaining the protective effects described in this paragraph (Stuebe & Rich‐Edwards, 2009). Further studies are needed to prove this hypothesis' validity.

Finally, breastfeeding has been shown to reduce both ovarian and breast cancer independent of pregnancy alone (Babic et al., 2020; Collaborative Group on Hormonal Factors in Breast Cancer, 2002). A recent pooled analysis integrating data from 13 case–control studies found that in single breastfeeding events, breastfeeding between 1 and 3 months led to an 18% decreased risk of ovarian cancer development, and breastfeeding for more ≥12 months was associated with a 34% lower risk (Babic et al., 2020). Protection was observed for overall ovarian cancer but also for the high‐grade serious subtype, which has the highest mortality. Another study integrated data from 47 epidemiological studies (from 30 countries) to investigate the protective effect of breastfeeding on breast cancer. A decrease by 4.3% of breast cancer was found for every 12 months of breastfeeding, in addition to a 7% decrease for each birth. No differences were observed between countries or based on other variables, including age, the number of births and the age at the first child's birth (Collaborative Group on Hormonal Factors in Breast Cancer, 2002).

## SOCIETAL AND PUBLIC HEALTH IMPLICATIONS

Various studies focused on different countries have evaluated the economic costs of lack of breastfeeding by estimating the cost of the avoidable morbidity and mortality attributable to breastfeeding, as well as the economic losses derived from the infant and mother mortality and cognitive losses (Bartick & Reinhold, 2010; Drane, 1997; Pretorius et al., 2021; Quesada et al., 2020). To summarise the global economic implication of breastfeeding, we are reporting the results from the ‘*Cost of Not Breastfeeding Tool*’, which was used to estimate the global impact that lack of breastfeeding following recommendations has from an economic point of view (Walters et al., 2019). Enough data was available to evaluate the number of annual childhood deaths from diarrhoea and pneumoniae, which could be prevented by following the WHO recommendations for breastfeeding, which was estimated to be 595,279. In women, an estimated 98,243 deaths from breast cancer, ovarian cancer, and T2D could also be prevented with breastfeeding. They estimated that inadequate breastfeeding might be responsible for 974,956 cases of childhood obesity every year. From an economic point of view, it is estimated that US$1.1 billion per year is the cost attributed to the preventable morbidity and mortality incurred by mothers and their children. US$53.7 billion is estimated to be lost in future earnings per year due to premature child and women's mortality, and US$285.4 billion due to cognitive losses (Walters et al., 2019). The degree of economic and life losses is variable among countries. For instance, childhood mortality is more prevalent in low‐income countries while a higher proportion of preventable maternal deaths is observed in uppermiddle‐income countries (Walters et al., 2019).

As per the latest Global Breastfeeding Collective Scorecard, in 2023, breastfeeding was initiated within 1 h after birth for 46% of new‐borns, and 48% of infants are breastfed up to 6 months of age (Collective, 2023). The target set by the Collective, which is led by UNICEF and WHO, is to reach a global rate of 70% for exclusive breastfeeding in the first 6 months of life by 2030. To achieve such goals, intervention at multiple levels is needed to encourage and support breastfeeding. Interventions that have been shown to be effective include education and breastfeeding support before, at and after delivery, involving not only the mother but also fathers, other family members and the wider community (Rollins et al., 2016). The International Labour Organisation standards for maternity leave are reached in only 23% of countries, leading to inadequate maternity protection given to millions of working mothers, of which 80% are in Africa and Asia (Organization, 2014; Rollins et al., 2016). Policies that support breastfeeding are urgently needed, including workplace policies, removal of societal and structural barriers, regulations for the marketing of breastmilk substitutes and other financial interventions to support breastfeeding. Ultimately, investment to improve breastfeeding rates would have beneficial effects on health outcomes for both mothers and their infants, as well as the wider global economy.

## FUTURE DIRECTIONS AND RECOMMENDATIONS

The beneficial impacts of breastfeeding spans mother‐infant health and wider society, but further research is warranted. While some evidence is based on large epidemiological studies, the results are ‘associations’ that may be confounded and they also fail to disentangle cause or effect. Furthermore, multiple studies were retrospective, thus subjected to recall bias, which may further explain inconsistencies between studies. Many of the health conditions discussed in this review are multifactorial and a genetic component is also prevalent, partially explaining the differences reported between countries. Future large‐scale longitudinal studies would be beneficial in understanding lifelong health implications of breastfeeding on infants and their mothers.

A mechanistic understanding of how human milk exerts its positive effects across the life course has not been determined, but likely involves microbial‐ and immune‐modulation. Given that not all mothers are able to breastfeed, research on the role of human milk bioactive components is key to improving maternal human milk alternatives. Furthermore, differences in human milk bioactive composition between mothers can impact infant disease risk, which may explain why breastfed infants can still develop the diseases discussed in this review. Thus, personalised supplementation of specific human milk bioactive components may also be important for infants who do receive maternal human milk. Overall, a more comprehensive understanding of microbiome‐dependent or independent mechanisms is critical to enabling the development of precision therapies.

## AUTHOR CONTRIBUTIONS

**Andrea C. Masi:** Writing – original draft; conceptualization; visualization; resources. **Christopher J. Stewart:** Conceptualization; funding acquisition; writing – original draft; supervision.

## CONFLICT OF INTEREST STATEMENT

CJS declares lecture honoraria from Nestlé Nutrition Institute. The other authors have no relevant conflicts of interest to disclose.

## Data Availability

Data sharing not applicable to this article as no datasets were generated or analysed during the current study.
